# Lanolin as a Natural Agent for Improving Hydrophobicity and Biological Durability of Wood

**DOI:** 10.3390/ma19122456

**Published:** 2026-06-08

**Authors:** Wojciech Ł. Grześkowiak, Martyna Wienke

**Affiliations:** Faculty of Forestry and Wood Technology, Poznan University of Life Sciences, ul. Wojska Polskiego 28, 60-637 Poznan, Poland; martynawienke46@gmail.com

**Keywords:** lanolin, wood protection, hydrophobization, preservation, impregnation, treatment, fungi, biological durability

## Abstract

As a society, we are facing an environmental crisis, and as a result, nature-based and environmentally friendly solutions are gaining increasing popularity. The development of environmentally friendly wood-protection systems is an important challenge in materials science. In this study, lanolin-based emulsions were systematically evaluated as natural agents for improving the hydrophobicity and biological durability of wood. Scots pine (*Pinus sylvestris* L.) samples were treated with four types of lanolin emulsions, including variants containing boric acid, and subsequently analysed in terms of contact angle, resistance to wood-decay fungi (*Coniophora puteana* and *Pleurotus ostreatus*), and susceptibility to mould and microfungi growth (*Chaetomium globosum*; and mixture of: *Chaetomium globosum*, *Aspergillus niger*, *Penicillium*, *Paeciliomyces variotti*, *Alternaria tenuis*, and *Trichoderma viride*). This study investigates whether, and to what extent, the application of lanolin affects surface hydrophobization and thus improves its resistance to fungi. The results demonstrate that lanolin treatments, like paraffin or carnauba wax, significantly increase surface hydrophobicity, with contact angles rising from approximately 58° for untreated wood to 85–105° for treated samples. This effect is associated with reduced biological degradation, as evidenced by lower mass loss in treated samples compared to controls. Depending on the formulation, mass loss was reduced by up to approximately 30 percentage points for *Coniophora puteana* and up to approximately four percentage points for *Pleurotus ostreatus*. The incorporation of boric acid further enhanced resistance to wood-decay fungi, while slightly reducing contact angle values. The results indicate that lanolin-based emulsions can effectively improve both the moisture resistance and the biological durability of wood. The study provides a comprehensive experimental assessment of lanolin as a sustainable alternative to conventional hydrophobic agents and demonstrates its potential for application in wood-protection systems.

## 1. Introduction

Water, a fundamental factor in sustaining life on Earth, is also a stimulant for the growth of wood-feeding microorganisms. It supports the wood-decaying process and, at the same time, shortens the lifetime of wooden products. As a consequence of this process, moisture protection is a main challenge in wood processing [1,2].

**Figure 1:**
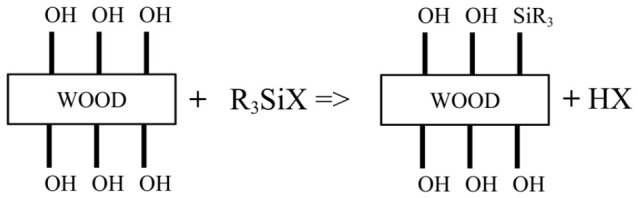
Scheme of sillilation of wood.

Among methods for wood surface modification aimed at increasing its hydrophobicity, we can distinguish sililation [2], which involves attaching silicone groups to the wood surface via covalent bonds (Figure 1). This process allows for the formation of a superhydrophobic, silicone-solvent-resistant surface [3]. As Broda [4] stated, finishing wood with organosilane compounds might be used in preservation work. The potential is largely determined by the interaction between organosilane compounds and functional groups on the wood surface, which strengthens the cell wall structure by filling voids and forming a stabilising layer on the surface. As a consequence of this interaction, the hygroscopicity of wood decreases, thereby reducing its potential for biodegradation [4]. Organosililation of thermally modified pine wood improves its resistance to *Coniophora puteana* by reducing the samples’ mass loss by 46–88% compared to unmodified samples [5].

Wood hydrophobization can also be achieved using natural finishing products, such as waxes and fats. In the category of well-known products are beeswax, linseed oil, and linseed oil varnish; on the other hand, we have products that are gaining popularity, such as carnauba wax [6,7]. Inspiration for achieving a superhydrophobic surface is largely drawn from lotus leaves. They are covered with a thick layer of wax crystals, which, thanks to their nano- and microstructural roughness, reduce the surface of contact between a leaf and a droplet of water. This type of structure causes the contact angle to be over 150 degrees [8,9,10].

The main disadvantages of using natural waxes in wood protection are high product cost and unstable properties that reduce hardness, adhesion, and thermo-resistance. As an alternative to natural waxes, Zhang and Song [11] recommended waxes synthesised using the Fischer–Tropsch method. At 140 °C, interfacial bonds form between modified Fischer–Tropsch waxes and wood, resulting in strong adhesion and high hardness by leveraging the wood’s inherent thermal stability [11]. Covering wood with a mixture of tung oil and beeswax, followed by deposition of micronised sodium chloride particles, resulted in the formation of a superhydrophobic layer [1].

Emulsions based on paraffin wax improve wood resistance against wood-decay fungi by increasing its hydrophobicity. As a result, the mass loss of protected wood samples is visibly lower than that of the control group [12]. Similar properties were observed on bamboo samples finished with a mixture of paraffin, microcrystalline wax, and stearic acid [13]. The presence of saturated fatty acids C5–C10 in wood-protection products might decrease the growth of moulds up to 12 weeks [14]. As a result of creating a hydrophobic layer, the addition of montane wax to the boric acid salt solution decreases the elution of boric acid from the tested samples by up to 50% [15].

Lanolin as a resource was already known and used in ancient times. At the beginning, it was thought that sheep wool wax was only a by-product of wool fabric production. However, many anthropologists hold that the extraction of lanolin by boiling wool was already known in the Bronze and Iron Ages. This perspective excludes the theory that lanolin is a by-product. Wool treated at high temperatures (e.g., in a boiling process at 100 °C) results in felted wool, which has no application in the textile industry. Wool for textile production requires spinning and weaving, which are impossible with boiled wool because it felts. Even in modern times, those two purposes of wool are treated differently in processing. Thanks to advances in chemical methods, lanolin is extracted using fat solvents or water-based scouring solutions [16,17].

Within the wool fat, two fractions can be separated: oxidised and unoxidised. The oxidised fraction is considered the fat from the tip of the wool fibre, which was exposed to air and characterised by a higher density than the unoxidised fraction [18]. Lanolin is an organic substance dissolved in organic solutions. During prolonged heating in an alcoholic solution of KOH or a glycerol solution of KOH, lanolin breaks down to cholesterol and potassium salts of fatty acids [19]. As a material, it is often compared to beeswax. With its emollient and humectant properties, it protects covered materials from both water gain and loss [16]. It creates emulsions with water in a minimal concentration of C = 20% (m/m) [19]. Lanolin is relatively easy to find and purchase in chemical supply stores; common packaging varies from 0.1 kg to 1.0 kg.

As many organic substances, i.e., paraffin, fatty acids, or glycerol, are used as phase-change materials, opening the door for lanolin. The most important factors that make paraffin an outstanding organic PCM are: high combustion temperature, low cost, rapid melting, and chemical stability [20]. There is a significant gap in the literature on the use of lanolin in wood protection. This paper aimed to investigate whether lanolin, as a potential phase-change material, can serve as a natural, ecological agent for protecting wood against biotic factors.

## 2. Materials and Methods

### 2.1. Materials

#### 2.1.1. Wood

Samples of pine wood (*Pinus sylvestris* L.), sapwood without defects, with a density of 588 kg/m^3^, cut out from one part of a log, were used for this experiment as a reference model of coniferous wood. The wood for the study was obtained from the Wielkopolska region. This tree is native to Europe and North Asia, where it constitutes approximately 2/3 of the wooded area in Poland. As a species, it requires a bright location, although it is used to growing in low-temperature zones. Pine could be classified as a “fat species”, thanks to its higher content of glycerides of fatty acids, which is responsible for its natural resistance against pests and diseases. On its radial section, there are 1–3 layers of thick-walled, folded tracheids next to the wood rays. Average density fluctuates around 550 kg/m^3^ [21,22,23].

The samples were air-conditioned for 2 months at 23 ± 2 °C and a relative humidity of 50 ± 5%, until they reached a constant weight. The average moisture content of samples measured with the drying-weighing method was 5.66%.

#### 2.1.2. Lanolin and Its Emulsions

Lanolin consists of a mixture of esters, unsaturated alcohols and higher carboxylic acids; however, it does not contain glycerol [18]. Sheep wool wax is built of fatty acids, cholesterol, alcano-1,2-diols, triterpene alcohols and trace amounts of hydrocarbons. Lanolin contains up to 138 fatty acids, of which alfa-hydroxylic and omega-hydroxylic fatty acids are the majority. It is a scentless substance with a characteristic yellow colour [19].

For this research, lanolin from the company AKTYN (Bielsko-Biała, Poland) was used; CAS: 8006-54-0, LOT: 05.2026. Preparation involved 4 different variants of lanolin emulsion, with water or a water-based boric acid solution. Boric acid (CHEMPUR, Piekary Śląskie, Poland, CAS: 10043-35-3) was used as a fungicide. Below are variants of prepared emulsions:

Symbol—components of emulsion—proportion in mass percentage:

- 50—lanolin:water—50:50.
- 20—lanolin:water—20:80.
- 50 kw—lanolin:4% water solution of boric acid—50:50.
- 20 kw—lanolin:4% water solution of boric acid—20:80.

Deposition of emulsion on a sample: 200 g/m^2^.

#### 2.1.3. Preparation of Emulsions

Based on previous tests, the mechanical agitator and the magnetic stirrer (as a heat source) were chosen for preparing lanolin emulsions. Lanolin liquefaction was tested at temperatures of 40, 50, and 60 °C, of which 60 °C was selected. This decision was based on lanolin’s appropriate melting point and the significantly shorter preparation time for a 50% emulsion at this temperature compared with lower temperatures.

Weighted lanolin was put in a beaker and then liquefied on a (magnetic) stirrer at a temperature of 60 °C. Afterwards, the mechanical agitator was placed in a beaker and set to 600 rpm. Liquid phase was added in equal parts. After every part, wait until the emulsion in the beaker becomes homogeneous.

#### 2.1.4. Preparation of Samples with Chosen Emulsions

Pine samples without defects were marked, measured, and weighed. Samples had an average moisture content of 5.66%. For contact angle samples, only 1 side was covered. Samples for all of the fungi resistance tests were finished with emulsion on all surfaces. Before applying it to the samples, the emulsion was reheated to 60 °C to melt it. Lanolin emulsions were applied using a glass rod.

Out of each variant of samples (same symbols as used for emulsions), 2 groups were separated due to the drying process:

- S—drying in a drier for 24 h at a temperature of 60 °C.
- L—drying in the laboratory for a period of 3 weeks, then followed by 24 h in a drier at a temperature of 40 °C.

Samples for fungi-resistance tests were dried after finishing each surface.

Planned deposition of emulsion on a sample: 200 g/m^2^ (Table 1). For each type of sample and its surface, the following range of emulsion deposition was set [g]:

- Contact angle: 0.300–0.340.
- Vulnerability to overgrowth by mould fungi and microfungi—upper/lower surface: 0.300–0.340.
- Vulnerability to overgrowth by mould fungi and microfungi—side surfaces: 0.140–0.180.
- Resistance against wood-decay fungi—upper/lower surface: 0.130–0.170.
- Resistance against wood-decay fungi—side surfaces: 0.110–0.150.

All measurements were performed in replicate, and the experimental design was structured to ensure repeatability and consistency of the results across independently prepared samples. The number of replicates varies across methods and is specified directly for each method in Section 2.2.

### 2.2. Methods

#### 2.2.1. Contact Angle

Contact angle is the angle between the surface of a solid object and the tangent to the surface of the liquid in contact with that solid. Measuring the contact angle is frequently used to determine surface hydrophobicity indirectly. As the contact angle increases, the surface water absorption capacity decreases, indicating increasing hydrophobicity [7].

Contact angle measurements were performed on the Goniometer DSA 25 KRÜSS—an analyser of droplet shape and surface tension (KRÜSS). A droplet with a volume of 1 mm^3^ was deposited onto the sample surface, and after 30 s, the contact angle was measured. From each variant, 3 samples were tested, and for each sample, the test was performed 3 times.

#### 2.2.2. Resistance Against Wood-Decay Fungi

Basidiomycota, known for decaying wood and other lignocellulosic materials, are a large fungal group found in buildings and wooden structures. For optimal growth, they need wood moisture in the range of 36–40%, a temperature of 18–27 °C, and a pH of 4–6.

The tests were conducted according to a modified procedure based on the EN 113-1:2020 standard [24]. According to the standard, two fungal species are required: *Conophora puteana* L. (brown rot) and *Trametes versicolor* L. (white rot). Due to the type of wood used, it was decided to use the required Cp fungus and, additionally, *Pleurotus ostreatus*, a representative of white rot, which also decays pine wood. The literature describes the possibility of modifying sample dimensions and shortening experimental time through the so-called mini-block test [23,24,25,26].

Due to the character of the emulsion—its melting at temperatures over 40 °C—samples covered with lanolin were not sterilised in the standard way in an autoclave before running an experiment. This kind of sterilisation process would increase the risk of damaging the emulsion and thereby decrease its hydrophobic properties. UV radiation was used for sterilisation, with samples exposed in a vaccination chamber for 24 h.

For testing, samples with dimensions of 50 × 15 × 5 [mm] were used. Each sample was measured and weighed. Half of the samples were finished with lanolin emulsions. Out of each composition (50, 20, 50 kw, 20 kw), in variants S and L, 10 samples were prepared. Half of each variant (Cp) was exposed to the activity of the fungus Coniophora puteana; the other half (P) was exposed to *Pleurotus ostreatus*. The malt-agar medium was prepared in the mass proportions agar:malt, extract:water, 2:5:100. After liquefying all ingredients, it was sterilised and poured into growth boxes. On a cooled-down and settled medium, the fungi were inoculated. Variant “Cp” stands for *Coniophora puteana* and “P” for *Pleurotus ostreatus*. Both control and test samples were put in pairs in the growth boxes on a glass pad. Due to the character of the emulsion—its melting at temperatures over 40 °C—samples covered with lanolin were not sterilised in the standard way in an autoclave before running an experiment. This kind of sterilisation process would increase the risk of damaging the emulsion and thereby decreasing its hydrophobic properties. UV radiation was used for sterilisation, with samples exposed in a vaccination chamber for 24 h. Due to the methods used to finish the samples, their dry mass before the experiment was calculated.

After termination of an 8-week experiment, samples were cleaned of fungi, weighed, dried to a dry mass, and weighed again. Based on those measurements, the mass loss was calculated.

#### 2.2.3. Vulnerability to Overgrowth by Mould Fungi and Microfungi

The effectiveness of fungicides against microfungi was examined by the block method. Wood samples of dimensions 40 × 40 × 5 [mm] were finished with lanolin emulsions. Out of each emulsion variant, 10 samples were prepared. Before preparation, malt-agar medium (in mass proportions: agar:malt and extract:water, 2:3:100) was sterilised and poured into the growth boxes. After the medium had cooled down and settled, control and test samples were placed and then infected with the test fungi. The following variants were prepared: A—*Chaetomium globosum*; and B—mixture of: *Chaetomium globosum*, *Aspergillus niger*, *Penicillium*, *Paeciliomyces variotti*, *Alternaria tenuis*, and *Trichoderma viride*. Evaluation of overgrowth extension was performed using a macroscopic method [27] (Table 1) at 5, 10, 13, and 22 days of the experiment. Due to the character of the emulsion—its melting at temperatures above 40 °C—finished products were not sterilised before running the experiment, as stated in point 2.2.2.

**Table 1:** Macroscopic scale of evaluation of overgrowth [27].

Index	Extension of Overgrowth
3	Lack of mycelial growth on a sample: on the medium, there is an inhibition zone between the sample and the mycelium.
2	Lack of mycelial growth on a sample: on the medium, there is no inhibition zone between the sample and the mycelium.
1	Less than 1/3 of the sample surface was overgrown with the mycelium of the test fungi.
0	More than 1/3 of the sample surface was overgrown with the mycelium of the test fungi.
−1	The surface of the tested sample was significantly more overgrown than that of the control sample.

Statistical analysis was performed using one-way ANOVA at a significance level of *p* < 0.05 for all testing methods. No post hoc comparisons were performed.

## 3. Results

Planned deposition of emulsion: 200 g/m^2^, real average deposition of emulsion (Table 2).

### 3.1. Contact Angle

A droplet with a volume of 1 mm^3^ was deposited onto the sample surface, and the program was started. After 30 s, the contact angle was measured (Table 3). Statistical analysis of the contact angle results was performed using one-way ANOVA; *p*-value = 6.8 × 10^−19^; F test = 2.07.

**Figure 2:**
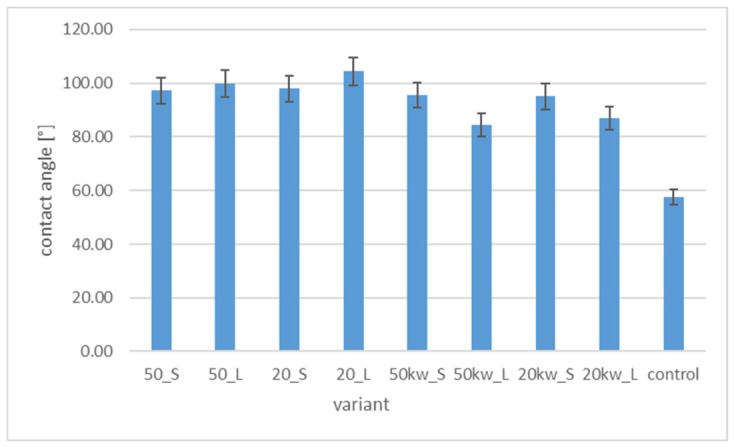
Results of measurements of contact angle for finished and control samples.

Contact angle measurements were performed on the tested samples, and the results were compared with those for the control samples. Based on Figure 2, all tested layer variants are characterised by higher contact angles than those of the unfinished wood surfaces.

**Figure 3:**
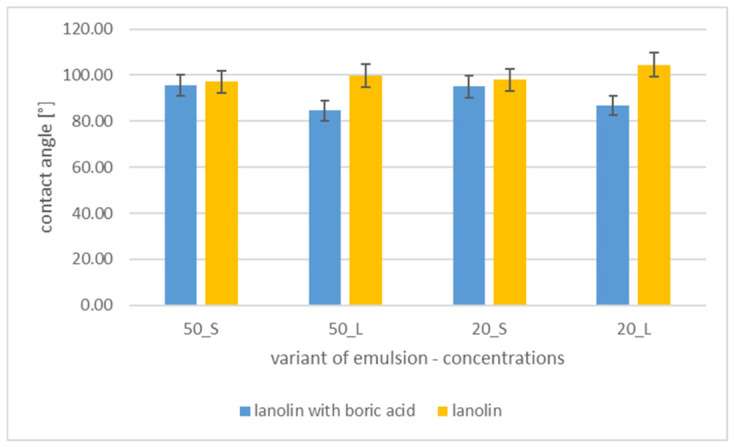
Contact angle for tested concentrations of lanolin emulsions with and without boric acid.

Figure 3 presents a comparison of the contact angles for the tested lanolin concentrations in variants with and without boric acid. It is visible that, regardless of lanolin concentration in the emulsion, the addition of boric acid causes a decrease in the contact angle. The observed differences between treated and control samples were consistent across all replicates, with variability remaining within the range indicated by the standard deviations, confirming the robustness of the experimental trends.

### 3.2. Resistance Against Wood-Decay Fungi

**Figure 4:**
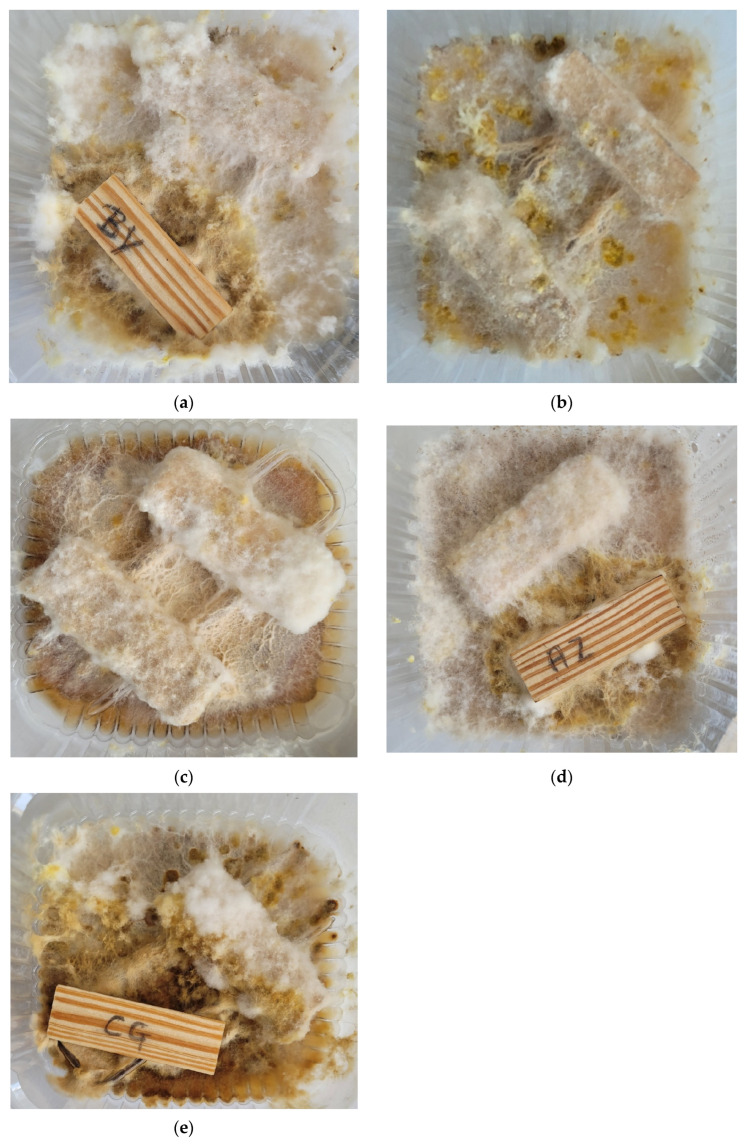
Samples exposed to activity of *Coniophora puteana*: (**a**) 50kw_L_Cp, (**b**) 50_S_Cp, (**c**) 50_L_Cp, (**d**) 50kw_S_Cp, and (**e**) 20kw_S_Cp (photography from the Author’s collection, 2025).

**Figure 5:**
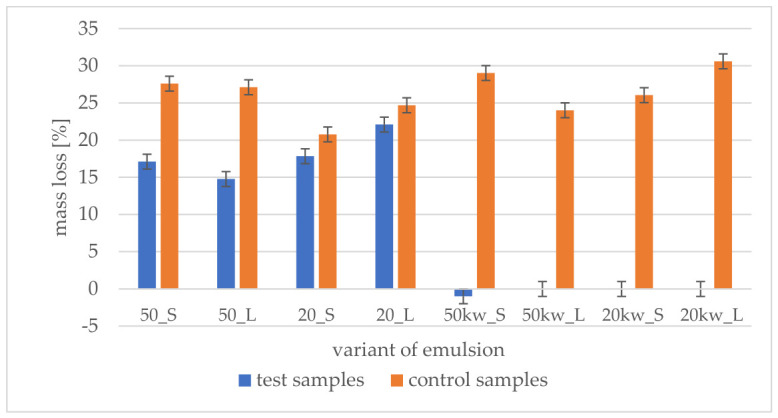
Average mass loss of samples exposed to the activity of *Coniophora puteana*. Negative values result from mycelial residues in the sample that were either hard or impossible to remove during brushing.

**Figure 6:**
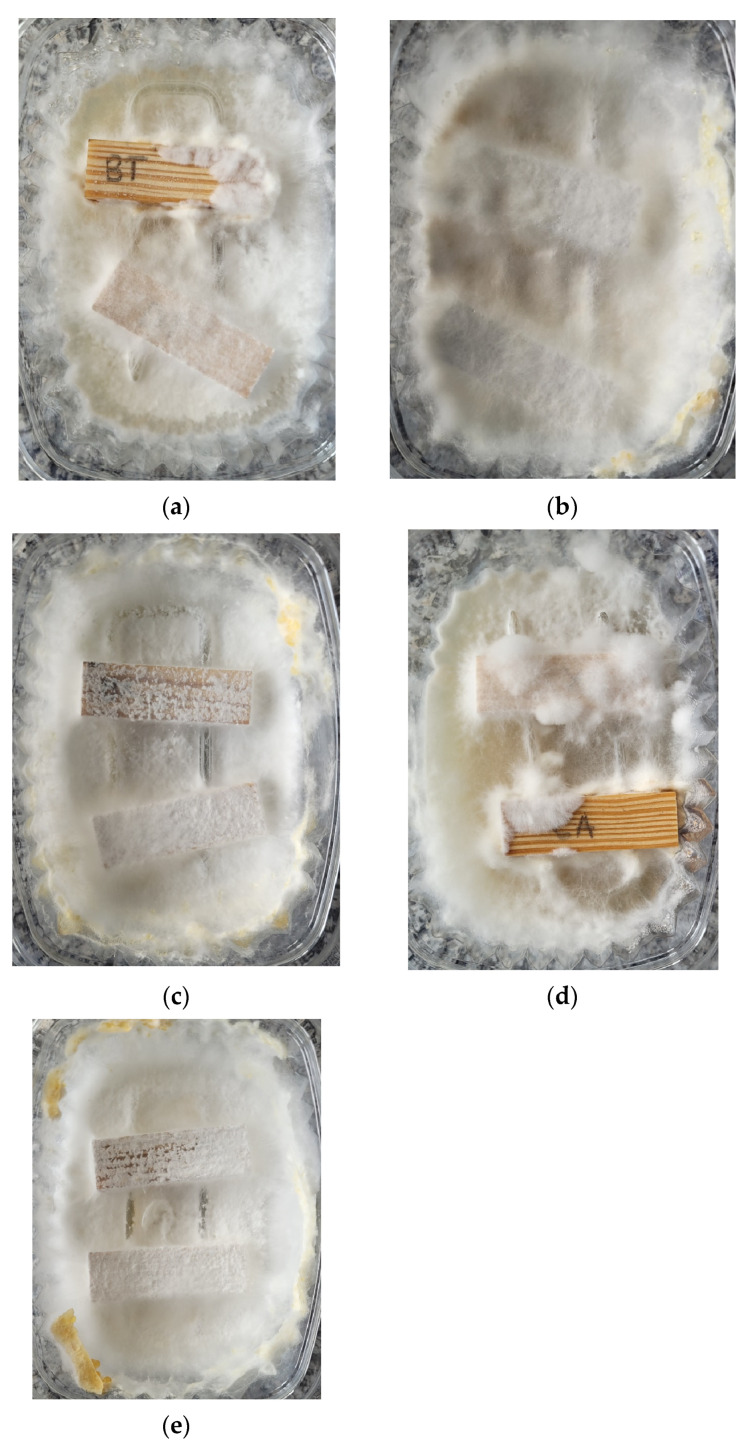
Samples exposed to the activity of *Pleurotus ostreatus*: (**a**) 50kw_L_P, (**b**) 20_S_P, (**c**) 20_L_P, (**d**) 20kw_S_P, and (**e**) 50_S_P (photography from the Author’s collection, 2025).

A total of 40 samples were exposed to the activity of fungi *Coniophora puteana* (variant Cp) (Figure 4 and Figure 5), and the other 40 (variant P) were exposed to *Pleurotus ostreatus* (Figure 6). The experiment was run in a grow room under stable conditions: t = 20 ± 2 °C and RH = 68%. Cultivation was terminated after 8 weeks, starting from the first observed mycelial growth. Then, the samples were weighed, dried to a dry mass, and weighed again. The percentage mass loss for the tested and control samples was analysed using the one-way ANOVA method (Table 4 and Table 5). The number of replicates used in the calculations varies across variants because of moulds; some samples were infected with moulds and had to be excluded.

Based on Figure 5, presenting the average mass loss of samples exposed to the activity of fungi *Coniophora puteana*, it was stated that in all emulsion variants, the mass loss is lower than in the case of control samples, which indicates that the mycelium growth was reduced. The most effective appeared to be emulsions with boric acid. Out of variants based only on lanolin and water, the best effects were shown by variant 50_L. The reduction in mass loss observed for treated samples was consistent across all tested variants, and the relatively low standard deviations indicate good measurement repeatability and the reliability of the observed effects.

**Figure 7:**
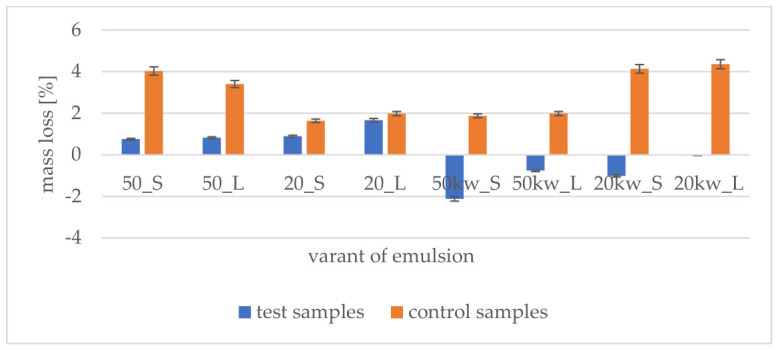
Average mass loss of samples exposed to the activity of *Pleurotus ostreatus*. Negative values result from mycelial residues in the sample that were either hard or impossible to remove during brushing.

From Table 5 and Figure 7, which show the average mass loss of samples exposed to *Pleurotus ostreatus* activity, it can be concluded that all emulsion variants reduced mass loss, reflecting the effectiveness of the finishes used. It is worth noting that, similar to Cp samples (Figure 5), the most effective variants were those with the addition of boric acid. The consistency of the results across replicates, together with relatively low standard deviations, indicates good data reproducibility and supports the validity of the observed trends. Both Figure 5 and Figure 7 show negative values. This indicates a mass loss of around 0.00%. Negative values result from mycelial residues in the sample that were either hard or impossible to remove during brushing. It can be assumed that in an extended experiment, mycelial residues would sustain the wood-decay process, resulting in further mass loss. Based on the experiment, the prepared variants significantly decrease the decay process.

**Figure 8:**
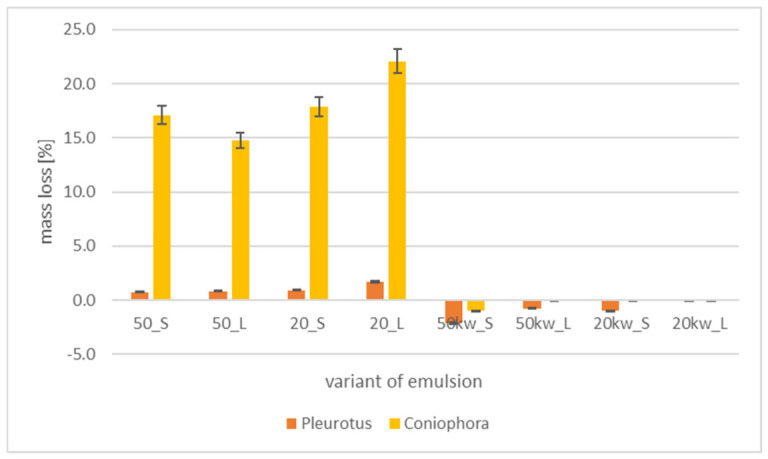
Mass loss of finished samples exposed to the activity of *Coniophora puteana* and *Pleurotus ostreatus*. Negative values result from mycelial residues in the sample that were either hard or impossible to remove during brushing.

In Figure 8, which compares mass loss among samples exposed to the activity of two wood-decay fungi, significantly greater mass loss is observed in samples exposed to *Coniophora puteana*. The decay character of the Pleurotus ostreatus mycelium might cause this phenomenon.

### 3.3. Vulnerability to Overgrowth by Mould Fungi and Microfungi

**Figure 9:**
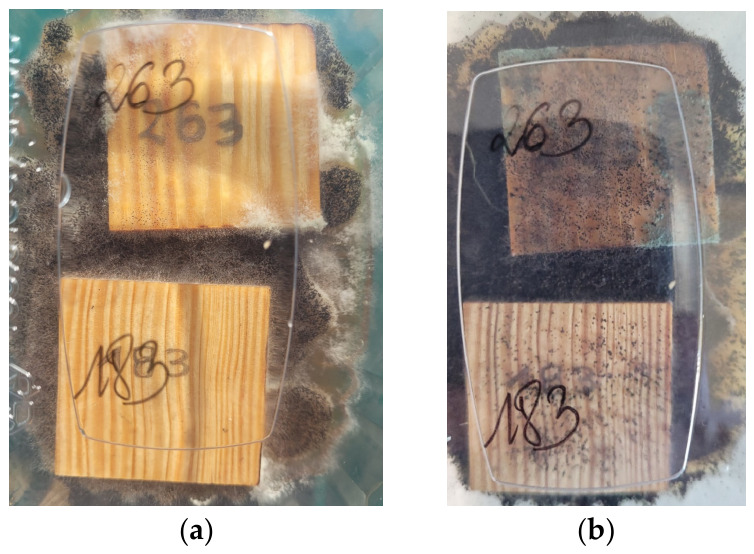

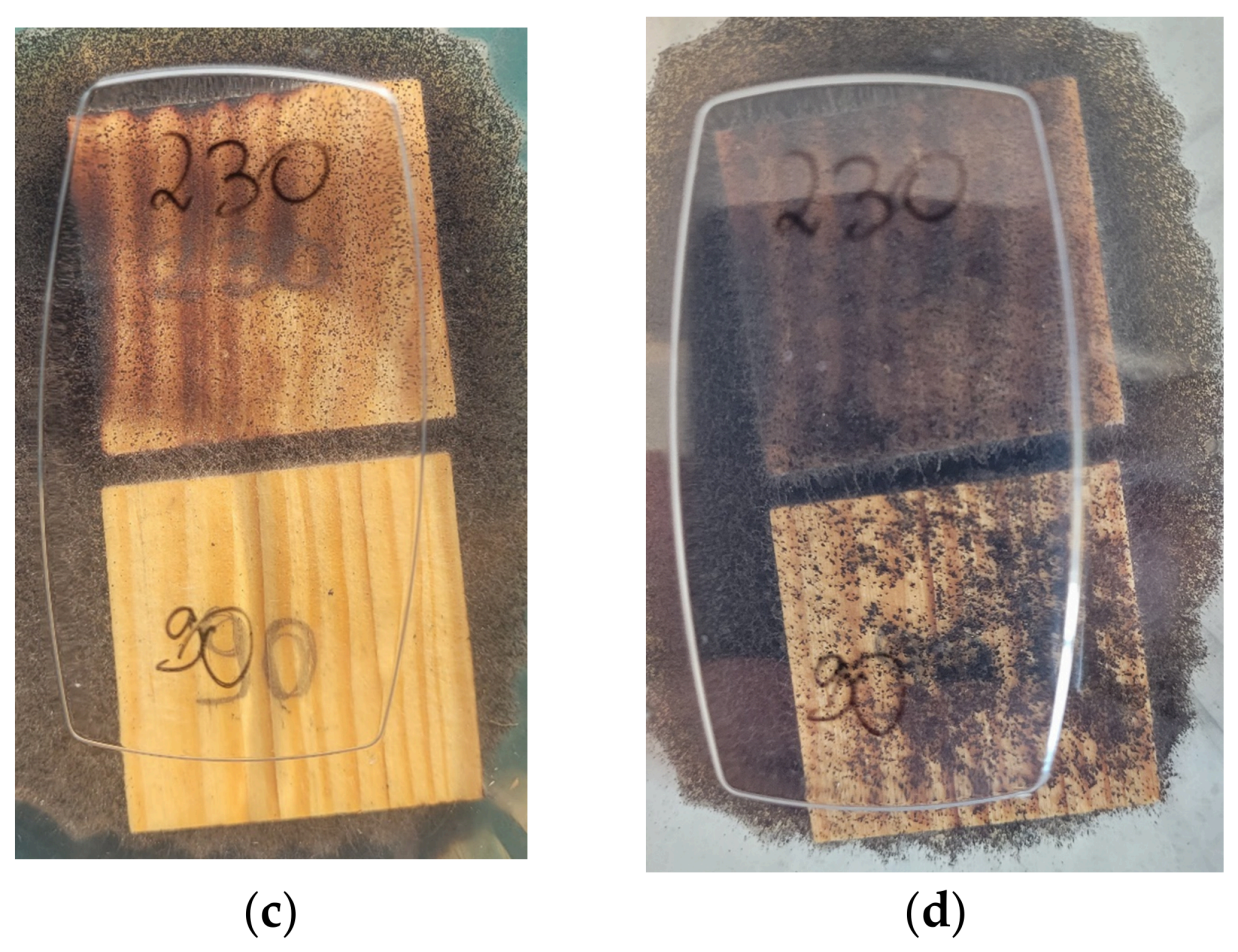
Samples exposed to the activity of moulds and microfungi: (**a**) 20kw_S_B after 5 days, (**b**) 20kw_S_B after 22 days, (**c**) 20_S_A after 5 days, and (**d**) 20_S_A after 22 days (photography from the Author’s collection, 2025).

**Figure 10:**
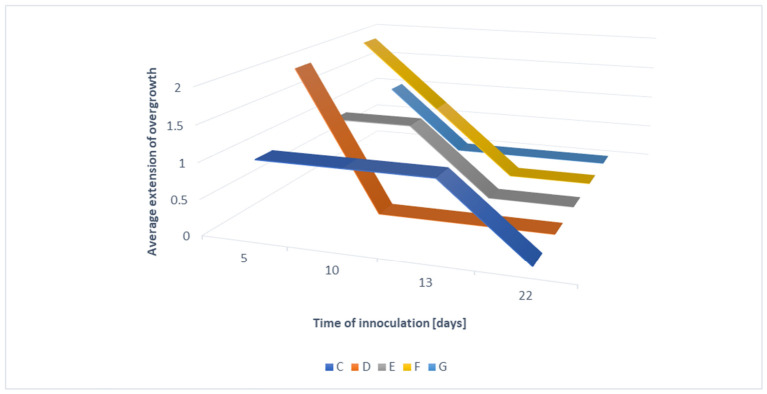
Evaluation of the average extension of overgrowth on samples exposed to moulds and microfungi.

A total of 40 samples were sprayed with spores of *Chaetomium globosum* (A) (Figure 9c,d); the other 40 were exposed to the activity of a mixture of *Chaetomium globosum*, *Aspergillus niger*, *Penicillium*, *Paeciliomyces variotti*, and *Alternaria tenuis. Trichoderma viride* (B) (Figure 9a,b). After 5, 10, 13, and 22 days, the extent of overgrowth in the samples was evaluated (Table 6; Figure 10). In Figure 10, variants were grouped into five categories (C, D, E, F, and G) based on their results in the evaluation of the average extension of overgrowth on samples by microfungi and moulds. Those groups were created to provide a clear graphical presentation of overgrowth trends observed during tests. Variants are categorised under certain groups:

- C: 50_S_B; 50_L_B; 20_L_B; 50kw_L_B.
- D: 50_S_A; 50_L_A; 20_S_A; 20_L_A.
- E: 20_S_B; 50kw_L_A; 20kw_S_A; 20kw_L_A.
- F: 50kw_S_B; 20kw_S_B; 20kw_L_B.
- G: 50kw_S_A.

## 4. Discussion

Waxes and fats are widely used in wood protection for their ability to reduce moisture uptake, thereby limiting biological degradation [6]. This mechanism primarily relies on increasing the hydrophobicity of the finished surfaces [1]. The process reduces the intensity of water absorption in wood, a key factor in wood biodegradation [1,2]. The results obtained in this study confirm that lanolin acts through a similar hydrophobization mechanism, forming a protective barrier on the wood surface.

The observed increase in contact angle (85–105°) indicates a significant reduction in surface wettability compared to untreated wood. This effect can be attributed to the presence of long-chain fatty acids, alcohols, and esters in lanolin, which create a low-surface-energy layer. As a result, water penetration into the wood structure is reduced, limiting the moisture available for fungal growth and metabolic activity. Since wood-decay fungi depend on moisture content typically above the fibre saturation point, a reduction in water uptake directly reduces the intensity of biodegradation.

Based on the research of Lesar [15], the incorporation of boric acid into the emulsions introduces an additional mechanism of protection. In this case, the system combines hydrophobic barrier formation with chemical biocidal action. While boric acid directly inhibits fungi, the presence of lanolin likely limits its leaching by reducing water penetration, resulting in a more sustained protective effect. This synergistic interaction explains the superior performance of lanolin–boric acid emulsions compared to lanolin-only systems. This mechanism is further supported by the observed decrease in mass loss in treated samples. Lower mass loss values indicate reduced fungal degradation, which can be directly linked to restricted moisture transport and reduced accessibility of cell wall components to enzymatic attack. The particularly strong effect observed for *Coniophora puteana* suggests that lanolin-based hydrophobization is especially effective against brown-rot fungi, which rely on diffusion of low-molecular-weight agents facilitated by moisture.

At the same time, the slight reduction in contact angle observed for boric acid-containing emulsions may result from increased polarity of the system and changes in emulsion structure. This indicates a trade-off between hydrophobicity and biocidal functionality, which should be considered in formulation design.

In contrast, no clear correlation was observed between the treatment parameters and resistance to the growth of moulds and microfungi. This may be due to these organisms’ lower moisture requirements and their ability to colonise surfaces even under limited water availability. Therefore, while hydrophobization effectively limits deep fungal decay, it may have a lesser impact on surface colonisation processes.

Overall, the results demonstrate that lanolin’s primary mechanism of action is surface hydrophobization and moisture exclusion, which indirectly suppresses biological degradation. When combined with boric acid, this mechanism is enhanced by biocidal activity, resulting in a multi-functional wood-protection system.

In addition to its chemical composition, the potential effectiveness of lanolin as a wood-protection agent may be related to its ability to form a hydrophobic barrier on the wood surface. This barrier is expected to reduce water penetration and limit moisture availability for fungal growth, thereby indirectly improving the biological durability of treated wood.

The differences observed between the tested variants and control samples were confirmed by one-way ANOVA (*p* < 0.05), indicating a statistically significant effect of the treatment.

## 5. Conclusions

- Lanolin emulsions show high stability at room temperature, without the addition of an emulsifier. Tested variants remained emulsified after storage in a laboratory for over 6 months.
- This study demonstrates that lanolin-based emulsions are effective agents for improving both the hydrophobicity and biological durability of wood. The applied treatments resulted in a substantial increase in contact angle from 58° to 85–105°, indicating enhanced surface water repellency compared to untreated samples.
- The improved hydrophobicity translated into increased resistance to biological degradation. Treated samples exhibited significantly lower mass loss when exposed to wood-decay fungi, particularly for *Coniophora puteana* (*p*-value = 5.52 × 10^−24^; F test = 1.83). The incorporation of boric acid into the emulsions further enhanced antifungal performance, confirming the synergistic effect between hydrophobic protection and biocidal action. In the case of exposure to *Pleurotus ostreatus*, the average mass loss for protected samples is around 0.00%, whereas for control samples it ranges from 24.01% to 30.62%.
- In contrast, the resistance to mould and microfungi growth showed no clear dependence on lanolin concentration or the presence of boric acid under the tested conditions, indicating that additional factors may influence these interactions.
- Overall, the results confirm that lanolin-based emulsions provide a viable and environmentally friendly approach to wood protection. The study establishes a consistent relationship between surface hydrophobization and biological durability, demonstrating the potential applicability of lanolin in sustainable wood-protection systems.

## Figures and Tables

**Table 2:** Real average deposition of emulsion on samples [g/m^2^].

Symbol of Emulsion	Contact Angle	Vulnerability to Overgrowth by Mould Fungi and Microfungi	Resistance Against Wood-Decay Fungi
50	204.81	200.15	198.23
20	200.85	206.53	198.33
50 kw	195.36	200.37	198.55
20 kw	199.33	199.53	197.06

**Table 3:** Results of contact angle measurements for finished and control samples; *p*-value = 6.8 × 10^−19^; F = 2.07.

Variant	Count	Sum	Average [°]	S^2^	SD
50_S	9	874.79	97.20	49.10	7.01
50_L	9	898.08	99.79	39.73	6.30
20_S	9	881.47	97.94	42.07	6.49
20_L	9	940.13	104.46	39.46	6.28
50_kw_S	9	860.67	95.63	102.22	10.11
50_kw_L	9	760.96	84.55	50.72	7.12
20_kw_S	9	855.79	95.09	137.88	11.74
20_kw_L	9	781.79	86.87	21.76	4.67
Control samples	9	518.40	57.60	96.41	9.82

**Table 4:** Results of mass loss for samples exposed to the activity of *Coniophora puteana*. *p*-value = 5.52 × 10^−24^; F test = 1.83; the number of replicates used for calculations varies between variants. It was necessary to eliminate samples infected by moulds. Negative values result from mycelial residues in the sample that were either hard or impossible to remove during brushing.

Variant	Tested Sample					Control Sample				
	Count	Sum	Average Mass Loss [%]	S^2^	SD	Count	Sum	Average Mass Loss [%]	S^2^	SD
50_S	4	68.45	17.11	10.74	3.28	4	110.42	27.61	62.11	7.88
50_L	5	73.89	14.78	30.16	5.49	5	135.60	27.12	26.34	5.13
20_S	5	89.22	17.84	9.69	3.11	5	103.84	20.77	8.48	2.91
20_L	4	88.40	22.10	9.47	3.08	4	98.78	24.70	2.15	1.47
50kw_S	5	−4.96	−0.99	0.03	0.18	5	145.14	29.03	15.94	3.99
50kw_L	5	−0.02	0.00	0.00	0.00	5	120.07	24.01	16.28	4.04
20kw_S	5	−0.02	0.00	0.00	0.00	5	130.27	26.05	11.22	3.35
20kw_L	5	−0.02	0.00	0.00	0.00	5	153.08	30.62	95.65	9.78

**Table 5:** Results of mass loss for samples exposed to the activity of *Pleurotus ostreatus*. The number of replicates used for calculations varied between variants. It was necessary to eliminate samples infected by moulds. Negative values result from mycelial residues in the sample that were either hard or impossible to remove during brushing.

Variant	Tested Sample					Control Sample				
	Count	Sum	Average Mass Loss [%]	S^2^	SD	Count	Sum	Average Mass Loss [%]	S^2^	SD
50_S	5	3.77	0.75	0.15	0.39	5	20.15	4.03	0.06	0.25
50_L	3	2.48	0.83	0.10	0.32	3	10.21	3.40	0.94	0.97
20_S	5	4.48	0.90	0.36	0.60	5	8.19	1.64	0.24	0.49
20_L	5	8.34	1.67	0.18	0.42	5	9.91	1.98	0.11	0.34
50kw_S	4	−8.47	−2.12	0.02	0.14	4	7.50	1.88	0.24	0.49
50kw_L	3	−2.28	−0.76	0.35	0.59	3	5.96	1.99	0.22	0.47
20kw_S	4	−4.06	−1.01	0.42	0.64	4	16.54	4.13	0.16	0.40
20kw_L	3	−0.09	−0.03	0.40	0.63	3	13.08	4.36	0.53	0.72

**Table 6:** Evaluation of an average extension of overgrowth on samples by microfungi and moulds.

Variant	Time of Inoculation [Days]			
	5	10	13	22
50_S_B	1	1	1	0
50_S_A	2	0	0	0
50_L_B	1	1	1	0
50_L_A	2	0	0	0
20_S_B	1	1	0	0
20_S_A	2	0	0	0
20_L_B	1	1	1	0
20_L_A	2	0	0	0
50kw_S_B	2	1	0	0
50kw_S_A	1	0	0	0
50kw_L_B	1	1	1	0
50kw_L_A	1	1	0	0
20kw_S_B	2	1	1	0
20kw_S_A	1	1	0	0
20kw_L_B	2	1	0	0
20kw_L_A	1	1	0	0

## Data Availability

The original contributions presented in this study are included in the article. Further inquiries can be directed to the corresponding author.

## Abbreviations

The following abbreviations are used in this manuscript:

50_S	50% concentration of lanolin, dried in a drier
50_L	50% concentration of lanolin, dried in the laboratory
20_S	20% concentration of lanolin, dried in a drier
20_L	20% concentration of lanolin, dried in the laboratory
50kw_S	50% concentration of lanolin in 4% water solution of boric acid, dried in a drier
50kw_L	50% concentration of lanolin in 4% water solution of boric acid, dried in the laboratory
20kw_S	20% concentration of lanolin in 4% water solution of boric acid, dried in a drier
20kw_L	20% concentration of lanolin in 4% water solution of boric acid, dried in the laboratory
Cp	Samples exposed to the activity of *Coniophora puteana*
P	Samples exposed to the activity of *Pleurotus ostreatus*
A	Samples exposed to the activity of *Chaetomium globosum*
B	Samples exposed to the activity of a mixture of: *Chaetomium globosum*, *Aspergillus niger*, *Penicillium*, *Paeciliomyces variotti*, *Alternaria tenuis*, and *Trichoderma viride*
